# Long-Term Efficacy and Safety of Evocalcet in Japanese Patients with Secondary Hyperparathyroidism Receiving Hemodialysis

**DOI:** 10.1038/s41598-019-42017-z

**Published:** 2019-04-23

**Authors:** Keitaro Yokoyama, Ryutaro Shimazaki, Masafumi Fukagawa, Tadao Akizawa, On behalf of Evocalcet Study Group, Yoshitaka Maeda, Kazue Ueki, Takayuki Fujii, Ryoichi Miyazaki, Hisanori Azekura, Hirotake Kasuga, Yoshiyuki Tomiyoshi, Takeaki Shinzato, Ryuji Iwashita, Kenji Takada, Akio Suda, Takashi Nagaoka, Mitsuru Yoshimoto, Masatomo Taniguchi, Hiroshi Ogawa

**Affiliations:** 1 Division of Nephrology and Hypertension, Department of Internal Medicine, The Jikei University School of Medicine, 3-25-8 Nishi-Shimbashi, Minato-ku, Tokyo, 105-8461 Japan; 2 R&D Division, Kyowa Hakko Kirin Co., Ltd., 1-9-2 Otemachi, Chiyoda-ku, Tokyo, 100-0004 Japan; 3 Division of Nephrology, Endocrinology and Metabolism, Department of Internal Medicine, Tokai University School of Medicine, 143 Shimokasuya, Isehara-shi, Kanagawa, 259-1193 Japan; 4 Division of Nephrology, Department of Medicine, Showa University School of Medicine, Namics 301, 4-24-51 Takanawa, Minato-ku, Tokyo, 108-0074 Japan; 5 Department of Nephrology, JA Toride Medical Center, Ibaraki, Japan; 6 Dialysis Center, Sanshikai Toho Hospital, Gunma, Japan; 7 Department of Nephrology, Seirei Sakura Citizen Hospital, Chiba, Japan; 8 Department of Internal Medicine, Fujita Memorial Hospital, Fukui, Japan; 9 Department of Nephrology, Sanaru Sun Clinic, Shizuoka, Japan; 10 Department of Internal Medicine, Kaikoukai Central Clinic, Aichi, Japan; 11 Department of Nephrology, Takagi Hospital, Fukuoka, Japan; 12 Department of Nephrology, Shinzato Clinic Urakami, Nagasaki, Japan; 13 Department of Nephrology, Ueyama Hospital, Kagoshima, Japan; 14 Department of Nephrology, Tsukuba Gakuen Hospital, Ibaraki, Japan; 15 Department of Internal Medicine, Suda Clinic, Tokyo, Japan; 16 Department of Hematology, Sagamihara Clinic, Kanagawa, Japan; 17 Department of Urology, Ohno Memorial Hospital, Osaka, Japan; 18 Department of Internal Medicine, Fukuoka Renal Clinic, Fukuoka, Japan; 19 Department of Internal Medicine, Shinseikai Daiichi Hospital, Aichi, Japan

**Keywords:** Phase III trials, End-stage renal disease, Haemodialysis

## Abstract

Secondary hyperparathyroidism (SHPT) is a common complication of chronic kidney disease (CKD), and as the disease progresses SHPT is associated with systemic consequences, termed CKD-mineral and bone disorder. Currently, cinacalcet is indicated for the treatment of SHPT; however, cinacalcet is associated with upper gastrointestinal adverse events. Evocalcet has been developed to address these issues, but the long-term safety and efficacy of evocalcet need to be evaluated. To more accurately reflect clinical practice, this phase 3, multicenter, open-label study was specifically designed without a cinacalcet washout period, and focused on those patients who switched from cinacalcet to evocalcet. A total of 137 SHPT patients undergoing hemodialysis were enrolled, of whom 113 switched from cinacalcet to evocalcet. The most frequent type of adverse drug reaction was decreased adjusted calcium. The incidence of gastrointestinal-related adverse events did not increase in a dose-dependent manner as the dose of evocalcet was increased. The percentage of patients achieving the target intact parathyroid hormone concentration increased from 40.9% to 72.3% with 52-week treatment. The corrected serum calcium and phosphorus levels remained largely unchanged throughout the study. The long-term safety and efficacy of evocalcet was confirmed using a clinically relevant intra-subject dose-adjustment strategy in SHPT patients undergoing hemodialysis.

## Introduction

Secondary hyperparathyroidism (SHPT) is a major and common complication that develops in chronic kidney disease (CKD) patients undergoing hemodialysis (HD)^1^. SHPT is a maladaptive response triggered by hypocalcemia, hyperphosphatemia, and active vitamin D deficiency, which in turn causes parathyroid cells to overproduce parathyroid hormone (PTH)^2^. Excess levels of PTH are associated with systemic toxicity, known as CKD-mineral and bone disorder, which represents the cardiovascular and bone diseases^2,3^. SHPT has typically been treated with the active form of vitamin D to reduce PTH levels; however, this type of treatment has been associated with elevations in serum calcium levels, causing arterial calcification^2^.

Cinacalcet, a type of calcimimetic agent, was developed to interact with calcium-sensing receptors (CaSR) as a positive allosteric modulator to suppress PTH secretion^4–6^. In addition to controlling PTH secretion, cinacalcet has been demonstrated to be useful for improving mortality risk and cardiovascular outcomes in clinical research^7,8^. While cinacalcet is an effective agent for the treatment of HD patients with SHPT, it is commonly associated with gastrointestinal (GI) symptoms that cause poor drug adherence and treatment discontinuation^9,10^. Moreover, even in patients who continue therapy, these adverse events can result in inadequate dosages of cinacalcet being administered.

Evocalcet is a novel calcimimetic developed as an equally efficacious, but more tolerable, alternative to the currently available calcimimetics for SHPT patients. In pre-clinical studies, evocalcet demonstrated efficacy in suppressing serum levels of PTH and with less-marked effects on the GI tract in animals^11^. Previous clinical trials have highlighted the dose-response relationship of evocalcet (from 1 to 12 mg) and safety in HD patients^12,13^. More recently, a 30-week phase 3 study indicated that 1–8 mg of evocalcet was non-inferior to cinacalcet, with approximately 70% of patients showing improvement in intact PTH (iPTH) levels while also reporting fewer GI adverse events^14^. These results were not inferior to the guidelines set by The Japanese Society for Dialysis Therapy (JSDT) for the management of SHPT in HD patients^15^.

The current 52-week phase 3 study examined the long-term safety and efficacy of once-daily oral administration of 1–12 mg evocalcet in SHPT patients undergoing HD. Additionally, patients who were currently receiving cinacalcet were switched to evocalcet so as to accurately reflect the clinical setting.

## Methods

### Ethics

This study was reviewed and approved by the institutional review board of each participating site (Supplementary Text S1) and conducted in compliance with the Declaration of Helsinki, Good Clinical Practice, and applicable regulations. This study was registered on ClinicalTrials.gov (NCT02549404, 15/09/2015) and JAPIC (JapicCTI-153015, 10/09/2015). All patients provided written informed consent.

### Patients

Patients were enrolled according to the following key inclusion criteria: iPTH >240 pg/mL only for patients who had not previously been treated with cinacalcet, or cinacalcet treatment for at least 2 weeks prior to screening; corrected calcium serum levels ≥8.4 mg/dL; provision of written consent; aged ≥20 years at the time of consent; and undergoing thrice-weekly HD for at least 12 weeks prior to screening.

Prior treatment with cinacalcet was permitted until the day before initiation of evocalcet treatment, and no washout period was required, although patients who commenced or had a change in cinacalcet administration 2 weeks prior to screening were excluded. Patients who had previously been treated with cinacalcet were exempt from the iPTH eligibility criterion of >240 pg/mL, but still underwent screening without a washout period to determine evocalcet starting dose.

Patients who received treatment with bisphosphonates, denosumab, or teriparatide within the 24 weeks before the screening; parathyroidectomy within 24 weeks before screening; or peritoneal dialysis within 12 weeks before screening, were excluded. Patients who changed the dose or dosing regimen of an activated vitamin D drug or its derivative, phosphate binder, or calcium preparation from 2 weeks prior to screening were excluded, while patients who changed dialysis conditions (dialysate calcium level, dialyzer, prescribed dialysis time, prescribed frequency of dialysis per week) from 2 weeks prior to screening were also excluded.

### Study design

**Figure 1:**
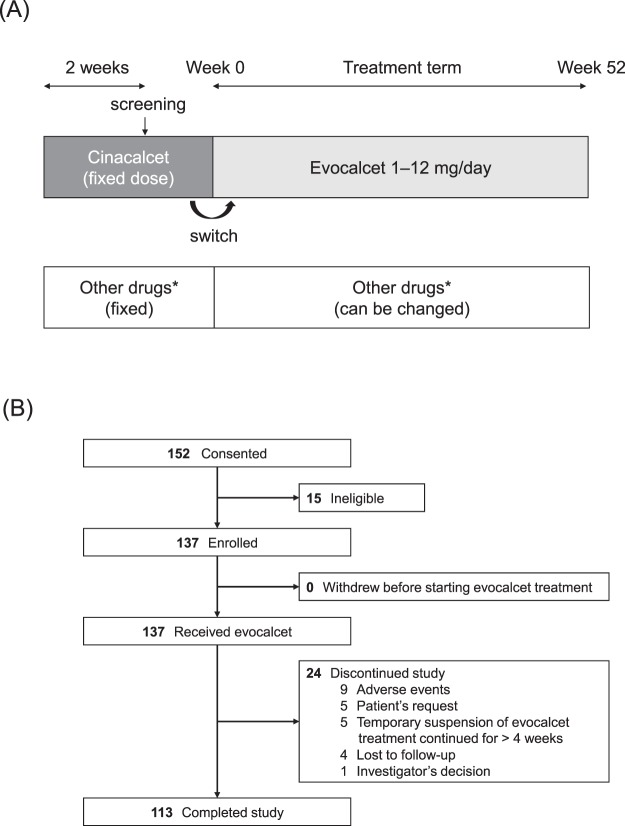
(**A**) Study design and (**B**) Disposition of patients. *Activated vitamin D drugs and their derivatives (calcitriol, maxacalcitol, falecalcitriol, alfacalcidol, and eldecalcitol), phosphate binders and calcium preparations (precipitated calcium carbonate, sevelamer hydrochloride, lanthanum carbonate, bixalomer, aluminum preparations, niceritrol, colestimide, colestyramine, ferric citrate hydrate, and others), and food with phosphorus binding effects (e.g. calcium acetate, eggshell calcium).

This was a phase 3, multicenter, open-label, intra-patient dose-adjustment study conducted in 15 participating centers (Supplementary Text S1) in Japan from September 2015 to December 2016. The study design is shown in Fig. 1A. After patients were screened, they were administered evocalcet orally once daily over a treatment period of 52 weeks. The dose-adjustment criteria aimed to maintain an iPTH concentration of 60–240 pg/mL and this was based on the target range proposed by the JSDT (Supplementary Text S2)^15^. The starting dose of evocalcet was nominally set at 1 mg, or 2 mg if patients had an iPTH concentration of ≥500 pg/mL and a corrected serum calcium concentration of ≥9.0 mg/dL at screening, regardless of pre-treatment cinacalcet dose. Treatment with cinacalcet, bisphosphonates, denosumab, or teriparatide; parathyroidectomy; and peritoneal dialysis were prohibited during the study. Changing or beginning an activated vitamin D drug or its derivative, phosphate binder, or calcium preparation was permitted throughout the study period. Further details on study design and exclusion criteria are summarized in Supplementary Text S2.

### Biochemical determinations

All clinical examinations were performed in centralized laboratories at LSI Medience Corporation. iPTH was measured using electro-chemiluminescence immunoassay (ECLusys PTH; Roche Diagnostics K.K., Tokyo, Japan). Intact fibroblast growth factor 23 (FGF23) was measured using enzyme-linked immunosorbent assay (FGF-23 ELISA Kit; KAINOS Laboratories, Inc., Tokyo, Japan).

### Safety analysis

Safety was primarily evaluated based on adverse events, adverse drug reactions, clinical laboratory values, vital signs, body weight, body mass index (BMI), 12-lead electrocardiogram (ECG), and ophthalmological examinations. Adverse drug reactions were defined as adverse events that were judged by an investigator to be causally related to evocalcet. Ophthalmological examinations and measurement of 12-lead ECG were performed before the administration of evocalcet and prior to HD at week 0, and then at any time needed thereafter. All other measures were performed before HD and evocalcet treatment after the maximum interdialytic interval. Adverse events and adverse drug reactions were coded by MedDRA ver. 19.0. Decreased adjusted calcium adverse events were reported when corrected serum calcium concentrations were ≤7.5 mg/dL or as judged by an investigator.

### Efficacy analysis

The efficacy endpoints included the proportion of patients with an iPTH concentration of 60–240 pg/mL, the percentage change in iPTH concentration from baseline, and the percentage of patients who achieved ≥30% decrease in iPTH concentration from baseline.

The median (interquartile range [IQR]) iPTH (pg/mL), whole PTH (pg/mL), intact FGF23 concentration (pg/mL), and bone turnover markers (bone-specific alkaline phosphatase [BSAP] [μg/L], tartrate-resistant acid phosphatase 5b [TRACP-5b] [mU/dL], and total procollagen type 1 intact N-terminal propeptide [P1NP] [μg/L]); and the mean (standard deviation [SD]) corrected serum calcium concentration (mg/mL), ionized calcium concentration (mEq/L), serum phosphorus concentration (mg/dL), and corrected serum calcium-phosphorus product (mg^2^/dL^2^) were also evaluated. Parathyroid volume and blood flow were assessed by carotid ultrasound.

### Statistical analysis

The sample size was based on the International Council for Harmonization of Technical Requirements for Pharmaceuticals for Human Use guidelines^16^. As a reference, a minimum of 100 patients was required for a 52-week study evaluating the long-term safety of evocalcet. Based on a previous long-term cinacalcet study in Japanese HD patients, a 15% discontinuation rate was calculated, and a target sample size of 120 patients was set^1^.

Categorical data were summarized as the frequency and percentage, and continuous data were summarized using descriptive statistics (the number of patients, median, IQR, mean, SD). The efficacy analysis was carried out using the full analysis set (FAS), which included all patients enrolled in the study who were administered evocalcet at least once and had iPTH levels measured at least once after the study began. The safety analysis was carried out using the safety analysis set, which included all patients enrolled in the study who were administered evocalcet at least once.

No statistical adjustment was made to missing data for continuous variables in the efficacy analysis of the present study. Missing data were treated as patients who did not achieve the target iPTH concentration of 60–240 pg/mL and, in order to show robustness, two different versions of the data (with and without missing data) are presented.

*Adhoc* analyses were conducted to evaluate the efficacy and dose of evocalcet in patients stratified by pre-treatment cinacalcet dose. The changes from baseline in iPTH, whole PTH, intact FGF23 concentration, and bone turnover markers were tested using Wilcoxon signed-rank tests. The percent change from baseline in iPTH and the changes from baseline in corrected serum calcium, ionized calcium, serum phosphorus, and corrected serum calcium-phosphorus concentration were assessed using t-tests. The number of patients who achieved target iPTH and patients who achieved a ≥30% decrease in iPTH from baseline were assessed using chi-squared tests.

## Results

### Patient background

Written informed consent was obtained from 152 Japanese HD patients with SHPT. Of these, 15 were judged to be ineligible for enrollment, and a total of 137 patients were enrolled in the present study to receive oral administration of evocalcet (Fig. 1B). The demographics of the enrolled patients are presented in Table 1. Of the 137 patients, 81 were male and 56 were female with a mean (SD) age of 60.3 (10.3) years. Of the 137 patients enrolled, 24 patients (17.5%) discontinued treatment and 113 patients (82.5%) completed the study up to week 52 (Fig. 1B). Of the 137 patients participating, evocalcet was taken as prescribed each day (adherence rate) 99.1 ± 2.2% of the time.

**Table 1:** Patient characteristics at baseline (*n* = 137).

Male, *n* (%)	81 (59.1)
Age, years	60.3 ± 10.3
Age, ≥65 years, *n* (%)	54 (39.4)
Dry weight, kg	58.7 ± 14.8
Body mass index, kg/m^2^	23.3 ± 4.2
Duration of dialysis, months	164 ± 115
Baseline cinacalcet use, *n* (%)	113 (82.5)
0 mg	24 (17.5)
12.5 mg	11 (8.0)
25 mg	46 (33.6)
37.5 mg	4 (2.9)
50 mg	28 (20.4)
75 mg	18 (13.1)
100 mg	6 (4.4)
Primary disease, *n* (%)	
Diabetic nephropathy	27 (19.7)
Chronic glomerulonephritis	67 (48.9)
Nephrosclerosis	4 (2.9)
Polycystic kidney disease	8 (5.8)
Chronic pyelonephritis	1 (0.7)
Other	30 (21.9)
Complications, *n* (%)	
Diabetes	36 (26.3)
Congestive heart failure	3 (2.2)
Long QT syndrome	2 (1.5)
Type of dialysis, *n* (%)	
Hemodialysis	90 (65.7)
Hemodiafiltration	47 (34.3)

Values are shown as *n* (%) or mean ± SD.

Of the 137 patients, 113 patients had the following cinacalcet treatment on the day before beginning evocalcet treatment (day −1): 12.5 mg (*n* = 11), 25 mg (*n* = 46), 37.5 mg (*n* = 4), 50 mg (*n* = 28), 75 mg (*n* = 18), and 100 mg (*n* = 6) cinacalcet. The remaining 24 patients did not receive the study drug on day −1. Concomitant therapy with activated vitamin D, calcium-based phosphate binders and calcium preparation is shown in Table 2.

**Table 2:** Concomitant therapy with activated vitamin D, calcium-based phosphate binders and calcium preparation.

	Baseline (*n* = 137)	Week 12 (*n* = 130)	Week 24 (*n* = 124)	Week 36 (*n* = 121)	Week 51 (*n* = 114)
Activated vitamin D (Injection)					
Maxacalcitol					
Patients, *n* (%)	55 (40.1)	54 (41.5)	53 (42.7)	53 (43.8)	52 (45.6)
Dose, µg/week	10.62 ± 7.86	13.80 ± 9.71	12.88 ± 8.08	14.67 ± 9.76	17.31 ± 10.90
Calcitriol					
Patients, *n* (%)	33 (24.1)	33 (25.4)	32 (25.8)	30 (24.8)	29 (25.4)
Dose, µg/week	2.11 ± 0.94	2.27 ± 1.08	2.19 ± 0.99	2.83 ± 1.40	3.33 ± 1.58
Activated vitamin D (Oral)					
Alfacalcidol					
Patients, *n* (%)	27 (19.7)	26 (20.0)	29 (23.4)	30 (24.8)	28 (24.6)
Dose, µg/day	0.35 ± 0.17	0.45 ± 0.21	0.40 ± 0.17	0.47 ± 0.27	0.55 ± 0.31
Calcitriol					
Patients, *n* (%)	4 (2.9)	4 (3.1)	3 (2.4)	3 (2.5)	3 (2.6)
Dose, µg/day	0.38 ± 0.14	0.38 ± 0.14	0.42 ± 0.14	0.58 ± 0.29	0.58 ± 0.29
Falecalcitriol					
Patients, *n* (%)	2 (1.5)	2 (1.5)	2 (1.6)	2 (1.7)	2 (1.8)
Dose, µg/day	0.23 ± 0.11	0.30 ± 0.00	0.30 ± 0.00	0.45 ± 0.00	0.53 ± 0.11
Calcium-based phosphate binders					
Calcium carbonate					
Patients, *n* (%)	81 (59.1)	82 (63.1)	79 (63.7)	81 (66.9)	73 (64.0)
Dose, mg/day	2204 ± 1129	2247 ± 1129	2503 ± 1341	2843 ± 1803	3380 ± 2271
Calcium preparation					
Calcium lactate					
Patients, *n* (%)	1 (0.7)	1 (0.8)	2 (1.6)	2 (1.7)	2 (1.8)
Dose, mg/day	2000 ± 0	3000 ± 0	4500 ± 2121	4500 ± 2121	4500 ± 2121

Doses are shown as mean ± SD and were calculated only from patients receiving concomitant drugs.

### Safety analysis

Adverse events occurred in 136 of the 137 patients (99.3%), and those judged to be moderate or severe in severity occurred in 29 patients and 14 patients, respectively (Table 3). The most frequent type of adverse drug reaction was decreased adjusted calcium (7.3%) (Table 3). Serious adverse events occurred in 41 patients (29.9%), and the most frequent type was shunt occlusion, which occurred in six patients (4.4%). Adverse drug reactions occurred in 48 patients (35.0%) with serious adverse drug reactions occurring in five patients (3.6%). These serious adverse drug reactions were congestive cardiac failure, cardiomyopathy, cataract, intestinal obstruction, and drug-induced liver injury.

**Table 3:** Adverse events (occurring in ≥5% of patients).

AEs	*n* (%)
Number of patients with AE	136 (99.3)
Nasopharyngitis	84 (61.3)
Contusion	22 (16.1)
Nausea	16 (11.7)
Diarrhea	16 (11.7)
Upper respiratory tract inflammation	13 (9.5)
Abdominal discomfort	12 (8.8)
Vomiting	12 (8.8)
Arthralgia	12 (8.8)
Shunt occlusion	11 (8.0)
Wound	11 (8.0)
Skin exfoliation	11 (8.0)
Decreased adjusted calcium	10 (7.3)
Pain in extremity	10 (7.3)
Headache	10 (7.3)
Stomatitis	9 (6.6)
Internal hemorrhage	9 (6.6)
Constipation	8 (5.8)
Shunt stenosis	8 (5.8)
Contact dermatitis	8 (5.8)
Back pain	7 (5.1)
Muscle spasms	7 (5.1)
Pruritus	7 (5.1)
Dental caries	7 (5.1)
Influenza	7 (5.1)
Cough	7 (5.1)

Values are shown as *n* (%). Abbreviation: AE, adverse event.

Coded by MedDRA ver. 19.0.

Adverse events and adverse drug reactions that resulted in a study drug dose reduction or withdrawal occurred in 29 (21.2%) and 22 (16.1%) of the 137 patients, respectively. The types of events were decreased adjusted calcium (the most frequent type), occurring in 10 patients (7.3%); decreased blood calcium, in four patients (2.9%); constipation and diarrhea, each in two patients (1.5%); and erosive gastritis, intestinal obstruction, nausea, vomiting, chest discomfort, and decreased blood parathyroid hormone, occurring in one patient each (0.7%).

Upper GI-related adverse events that occurred in ≥5% of patients included nausea (11.7%), abdominal discomfort (8.8%), and vomiting (8.8%) (Table 3). Upper GI-related adverse drug reactions that occurred in ≥5% of patients included nausea (5.1%) and abdominal discomfort (5.1%).

No adverse events resulted in death, and there were no clinically significant changes in the clinical laboratory values, body weight, vital signs, ophthalmological measures, and 12-lead ECG for all patients throughout the 52-week study period.

**Figure 2:**
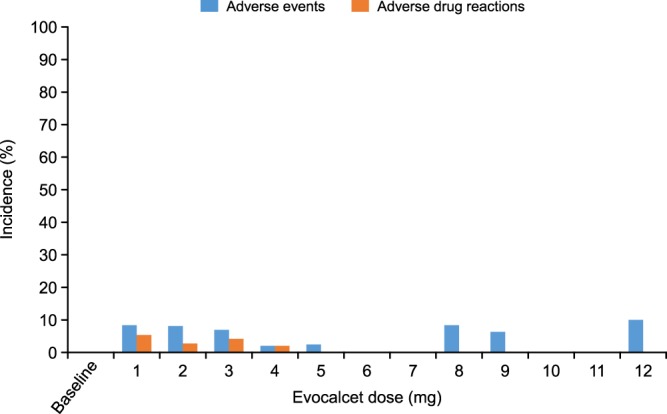
Incidence of GI-related adverse events and adverse drug reactions stratified by evocalcet dose at the first onset. GI-related adverse events were defined as a combination of nausea, vomiting, abdominal discomfort, decreased appetite, and abdominal distension. Abbreviation: GI, gastrointestinal.

The incidence of GI-related adverse events did not increase in a dose-dependent manner as the dose was increased from 3 mg to ≥8 mg evocalcet in 1-mg increments (Fig. 2). No new adverse events were observed with >8 mg evocalcet.

### Efficacy analysis

Of the 137 patients in the FAS, the number (%) of patients achieving an iPTH concentration of 60–240 pg/mL was 56 patients (40.9%) at baseline and 99 patients (72.3%) at week 52 (see Supplementary Fig. S1). When excluding those patients who discontinued the study (*n* = 24), the proportion of patients that achieved the target iPTH concentration at week 52 was 87.6% (99/113 patients). The target achievement rate also increased in the cinacalcet pre-treatment group from 49.6% (56/113 patients) at baseline to 70.8% (80/113 patients) at week 52 (Table 4).

**Table 4:** Efficacy parameters.

	Pre-treatment with cinacalcet		No pre-treatment with cinacalcet	
	Baseline (*n* = 113)	Week 52 (*n* = 93)	Baseline (*n* = 24)	Week 52 (*n* = 20)
Patients who achieved target iPTH concentration*^,^**, *n* (%)	56 (49.6)	80 (70.8)^‡^	0 (0.0)	19 (79.2)^‡^
Percent change in iPTH concentration from baseline, %	0	−4.2 ± 86.8	0	−64.9 ± 17.3^‡^
Patient who achieved ≥30% decrease in iPTH concentration from baseline**, *n* (%)	—	43 (38.1)^‡^	—	19 (79.2)^‡^
iPTH concentration, pg/mL	210 (143, 333)	152 (110, 202)^‡^	386 (310, 483)	135 (106, 156)^‡^
Whole PTH concentration, pg/mL	82 (53, 134)	74 (58, 92)	148 (119, 217)	69 (48, 85)^‡^
Corrected serum calcium concentration, mg/dL	9.16 ± 0.58	8.93 ± 0.59^‡^	9.04 ± 0.51	8.74 ± 0.46
Ionized calcium concentration, mEq/L	2.30 ± 0.18	2.32 ± 0.19	2.29 ± 0.10	2.33 ± 0.11
Serum phosphorus concentration, mg/dL	5.44 ± 1.17	5.12 ± 1.26^†^	5.31 ± 1.39	5.13 ± 1.05
Corrected serum calcium-phosphorus product, mg^2^/dL^2^	49.9 ± 11.3	45.7 ± 11.5^‡^	47.9 ± 12.1	45.0 ± 10.4
Intact FGF23 concentration, pg/mL	7700 (2510, 19500)	6520 (1990, 19300)	3040 (1073, 8685)	3315 (1665, 16200)
Bone turnover markers				
BSAP, μg/L	14.3 (11.1, 19.3)	13.7 (10.7, 16.9)	19.2 (11.9, 22.8)	10.7 (9.9, 13.1)^‡^
TRACP-5b, mU/dL	601 (430, 818)	420 (297, 644)^‡^	760 (586, 875)	314 (206, 437)^‡^
Total P1NP, μg/L	330 (217, 427)	224 (151, 313)^‡^	311 (250, 551)	146 (116, 214)^‡^

Values are shown as *n* (%), mean ± SD, or median (25^th^ and 75^th^ percentiles).

*Treatment goal recommended by JSDT: ≥60 pg/mL to ≤240 pg/mL, **Ratio was calculated dividing by *n* at baseline.

^†^p < 0.05, ^‡^p < 0.01.

Abbreviations: BSAP, bone-specific alkaline phosphatase; FGF23, fibroblast growth factor 23; iPTH, intact parathyroid hormone; IQR, interquartile range; P1NP, procollagen type 1 intact N-terminal propeptide; PTH, parathyroid hormone; SD, standard deviation; TRACP-5b, tartrate-resistant acid phosphatase 5b.

**Figure 3:**
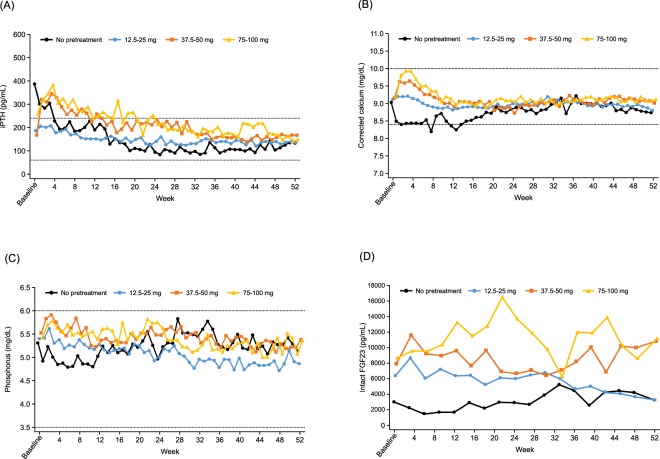
Time course of (**A**) iPTH, (**B**) corrected serum calcium, (**C**) serum phosphorus, and (**D**) intact FGF23 stratified by pre-treatment cinacalcet dose. Data are shown as median for iPTH and intact FGF23; and as mean for corrected serum calcium and serum phosphorus. Abbreviations: FGF23, fibroblast growth factor 23; iPTH, intact parathyroid hormone.

The mean percent change (SD) in iPTH concentration from baseline was −15% (82%) at week 52. The number (%) of patients who achieved a decrease in iPTH concentration of ≥30% from baseline was 62 (45.3%). The results of the *ad hoc* analyses show that, in patients who had not been previously treated with cinacalcet, the percentage change (SD) in iPTH concentration from baseline was −64.9% (17.3%), and the number (%) of patients who achieved a decrease in iPTH concentration of ≥30% from baseline was 19 (79.2%) (Table 4). In patients who had been treated with cinacalcet prior to the study, the iPTH concentration initially increased, then decreased with evocalcet dose adjustment (Fig. 3A). The number (%) of cinacalcet pre-treated patients who achieved a decrease in iPTH concentration of ≥30% from baseline was 43 (38.1%) (Table 4).

The mean (SD) corrected serum calcium concentration was 9.14 (0.57) mg/dL at baseline and 8.90 (0.57) mg/dL at week 52. Although the serum calcium concentration initially fluctuated, it remained almost unchanged throughout the study (Fig. 3B). The mean (SD) serum phosphorus concentration was 5.42 (1.21) mg/dL at baseline and 5.12 (1.22) mg/dL at week 52, also remaining almost unchanged throughout the study period (Fig. 3C).

The mean ionized calcium, corrected serum calcium-phosphorus product levels, and the median intact FGF23 also remained relatively unchanged (Fig. 3D and Table 4). The median concentrations of the bone turnover markers BSAP, TRACP-5b, and total P1NP decreased during the study period (Table 4 and Supplementary Figs S2–4). None of the indices of parathyroid volume showed a large change from baseline to week 52, and the same was true of the blood flow evaluation.

**Figure 4:**
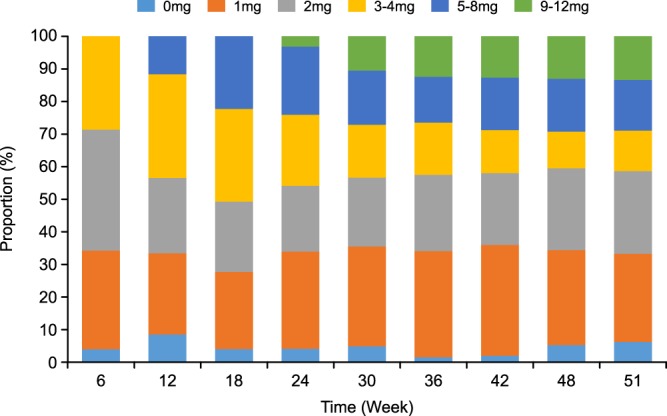
Proportion of patients in each dose category.

The median (IQR) evocalcet dose of all the patients increased from a baseline dose of 1.0 (0.0) mg to a final dose of 2.0 (4.0) mg with >50% of patients treated with ≤2 mg evocalcet (Fig. 4). The time courses of changes in evocalcet dose when stratified by pre-treatment cinacalcet dose (12.5–25 mg, 37.5–50 mg, and 75–100 mg) are presented in Supplementary Figure S5. The results of this *ad hoc* analysis show that the mean (SD) doses at week 51 in each subgroup were 1.7 (1.0) mg, 2.9 (2.6) mg, 4.8 (2.8) mg, 6.2 (3.9) mg, 5.4 (4.4) mg, and 10.3 (2.9) mg, respectively.

When stratified by pre-treatment cinacalcet dose (12.5 mg, 25 mg, 37.5 mg, 50 mg, 75 mg, and 100 mg), the evocalcet doses required to return the mean percent change in iPTH concentration to 0% were 1.09 mg, 1.82 mg, 1.75 mg, 5.52 mg, 4.50 mg, and 10.33 mg, respectively. Therefore, the ratios of pre-treatment cinacalcet dose divided by evocalcet dose were 11.5, 13.8, 21.4, 9.1, 16.7, and 9.7, respectively. Moreover, in the 40 patients who achieved an iPTH concentration of 60–240 pg/mL at both week 0 and 52, the mean ratio was 10.5. These ratios are consistent with a previous clinical trial, which showed that the efficacy of 2 mg evocalcet was nearly equivalent to that of 25 mg cinacalcet (ratio = 12.5)^17^. However, in some patients in the present study, a target iPTH level was only achieved after receiving an escalated evocalcet dose of 12 mg, which means that some patients had a ratio greater than the previously reported 12.5.

## Discussion

This was the first study to evaluate the long-term efficacy and safety of evocalcet in patients with SHPT undergoing HD, including patients that had switched from cinacalcet. Japanese patients have a longer duration of dialysis compared with patients in the US and EU; therefore, the development of a suitable therapy for Japanese patients with SHPT is needed^18^. Because switching from cinacalcet to evocalcet was unique to the design of the present study, and the safety of evocalcet in Japanese hemodialysis patients with SHPT has been already elucidated in another head-to-head phase 3 study^14^, this discussion focuses on the secondary endpoints of the present study.

In this study, the number of patients who reached the target range (iPTH concentration of 60–240 pg/mL) progressively increased with continued administration of evocalcet over the 52-week study period. The majority of patients (82.5%) were switched from cinacalcet to evocalcet; therefore, it was assumed that the baseline parameter values would have been influenced by the prior treatment with cinacalcet. As such, patients pre-treated with cinacalcet had a median iPTH concentration at baseline of 210 pg/mL, whereas in patients who were not pre-treated with cinacalcet, the median iPTH concentration was 386 pg/mL. This indicates that there was some level of improvement already accomplished by cinacalcet prior to this study. However, in patients who had been previously treated with cinacalcet, the iPTH concentration decreased further after switching to evocalcet. As a result, in these patients, the proportion of patients who achieved the target iPTH range increased from 49.6% (56/113 patients) at baseline to 70.8% (80/113 patients) at week 52. In patients who had not been previously treated with cinacalcet, the percentage change in iPTH concentration from baseline was −64.9%, and the number (%) of patients who achieved a decrease in iPTH concentration of ≥30% from baseline was 19 (79.2%). These iPTH target achievement results are comparable to those achieved with other calcimimetic agents^14,19–23^.

Although iPTH, calcium, and phosphorus levels temporarily increased after switching from cinacalcet, these fluctuations may be attributed to the pre-treatment cinacalcet dose as well as the relatively low evocalcet dose (1 or 2 mg) these patients were initially administered. To evaluate the impact of pre-treatment cinacalcet dose, we performed *ad hoc* analyses to evaluate the changes in these parameters according to pre-treatment cinacalcet dose. This analysis showed that patients pre-treated with a higher dose of cinacalcet had temporary increases in the efficacy parameters; however, these were then subsequently controlled with evocalcet dose adjustment. Additionally, the doses of activated vitamin D, calcium-based phosphate binders and calcium preparation tended to be increased throughout the study period to recover serum calcium levels that had decreased owing to the dose escalation of evocalcet. Overall, in all patient groups stratified by pre-treatment cinacalcet dose, the target iPTH achievement rate improved.

The data on FGF23, the bone turnover markers, and the results of the parathyroid volume analyses were inconclusive. However, this is believed to be due to the influence of pre-treatment with cinacalcet.

Evocalcet was generally well tolerated at the doses administered in this long-term study, particularly in regards to GI-related adverse events. Furthermore, there were no differences in the safety profiles between patients who were pre-treated with cinacalcet and those patients who were not. Most significant adverse events were deemed to be mild. Serious adverse drug reactions included congestive heart failure and cardiomyopathy; however, these occurred in only one patient each, and they either improved or were resolved with or without treatment. No significant events occurred due to a dose increase in evocalcet, which allows for dose escalation, further improving drug efficacy. Previous treatment with cinacalcet posed no safety concerns for patients in this study, and the incidence rates of adverse events were not affected by patients’ history of cinacalcet treatment.

Overall, the present study demonstrated that the safety profile of evocalcet supports the long-term administration with clinically relevant dosages. In some patients, a target iPTH level was only achieved after receiving an escalated evocalcet dose of 12 mg, which corresponds to a ratio that exceeds that of the previously reported 12.5. In these patients, the evocalcet dose was escalated to 12 mg because it was considered that these patients could not increase their pre-treatment cinacalcet dose sufficiently to achieve the necessary target iPTH. Additionally, baseline iPTH levels, intact FGF23 levels, parathyroid gland volume, and pre-treatment cinacalcet dose in these patients were higher than those reported for the overall average.

This study is limited by the unknown disease management status or compliance with cinacalcet treatment in those patients who had been treated with cinacalcet prior to the start of this study. Moreover, mineral and bone disorder markers might also be affected by concomitant therapy with activated vitamin D, calcium-based phosphate binders and calcium preparation. This study was also limited by not being placebo controlled, having a small number of patients, the possibility of attrition bias, and including only Japanese patients, which limits the generalizability of the results.

## Conclusions

This study demonstrated the long-term safety and efficacy of evocalcet in HD patients with SHPT over a 52-week period. The iPTH concentrations and the percentage of patients achieving the target range improved from baseline throughout the study after switching from cinacalcet. Evocalcet was well tolerated and the incidence of GI complications was low. Patients who had been pre-treated with cinacalcet were able to switch to evocalcet with no serious safety concerns observed.

## Supplementary information

Supplementary Information

## Data Availability

The datasets generated during and/or analyzed during the current study are available from the corresponding author on reasonable request.
