# Supplementary material for: Long-Term Efficacy and Safety of Evocalcet in Japanese Patients with Secondary Hyperparathyroidism Receiving Hemodialysis
Source: Sci Rep. 2019 Apr 23;9:6410. doi: 10.1038/s41598-019-42017-z (PMC6478860; doi:10.1038/s41598-019-42017-z)
Supplement: Supplementary file 1 — Supplementary Information [file 41598_2019_42017_MOESM1_ESM.pdf]

Long-Term Efficacy and Safety of Evocalcet in Japanese Patients with Secondary Hyperparathyroidism Receiving Hemodialysis

Keitaro Yokoyama, Ryutaro Shimazaki, Masafumi Fukagawa, Tadao Akizawa & Evocalcet Study Group

Supplementary Figure S1: Percentage of patients who achieved iPTH treatment target of 60–240 pg/mL

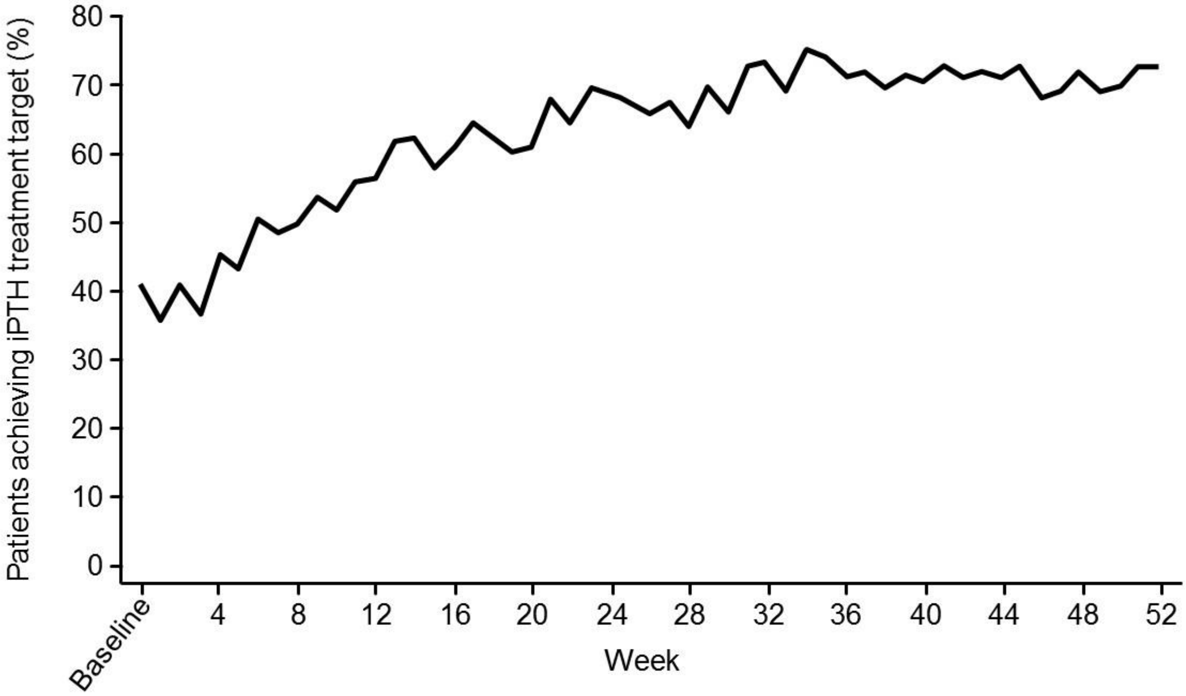

Abbreviation: iPTH, intact parathyroid hormone

Supplementary Figure S2: Time course of BAP

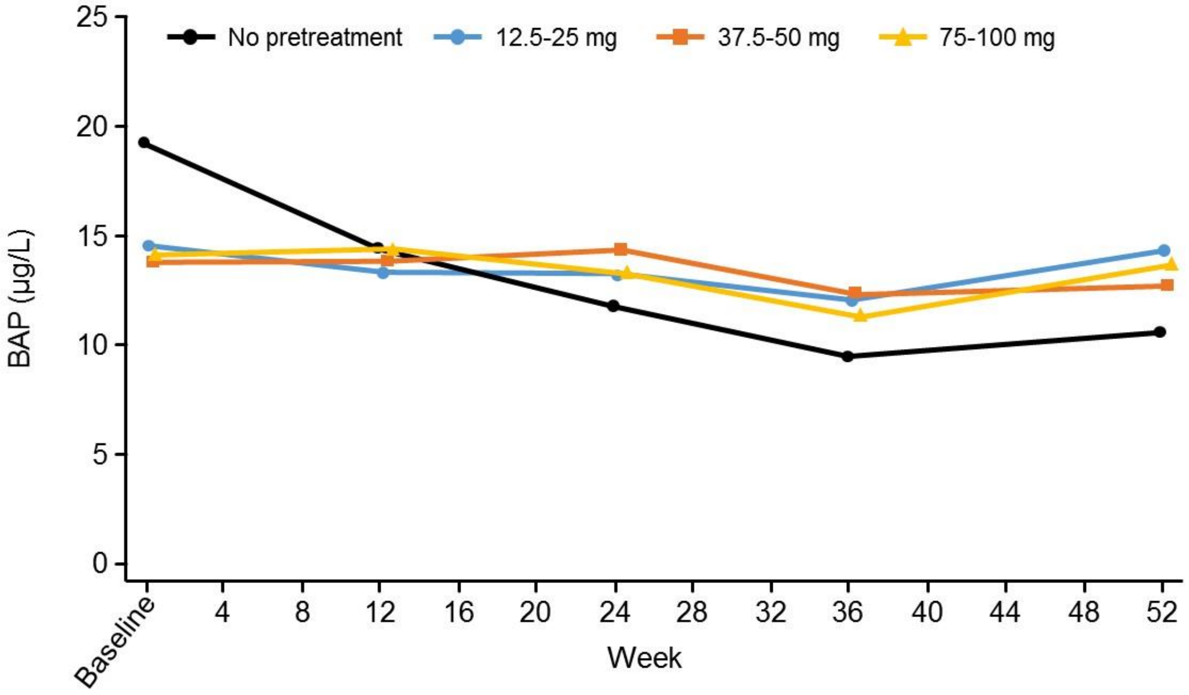

Data are shown as median.

Abbreviation: BAP, bone-specific alkaline phosphatase

Supplementary Figure S3: Time course of TRACP-5b

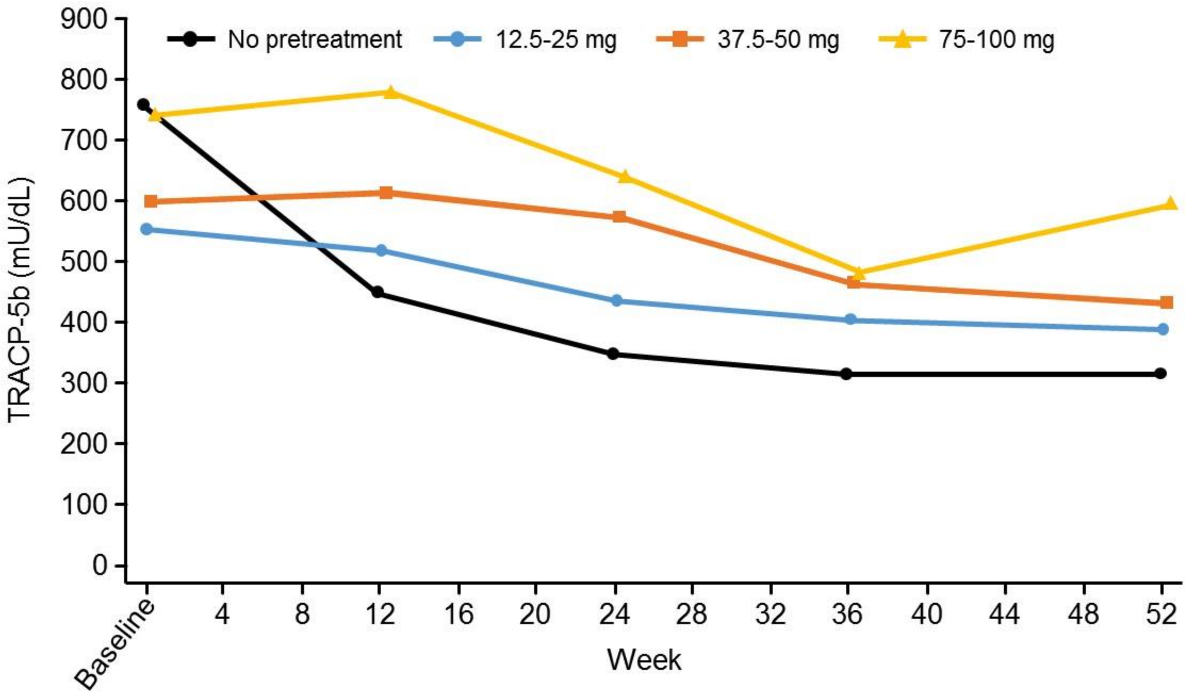

Data are shown as median.

Abbreviation: TRACP-5b, Tartrate-resistant acid phosphatase 5b

Supplementary Figure S4: Time course of total P1NP

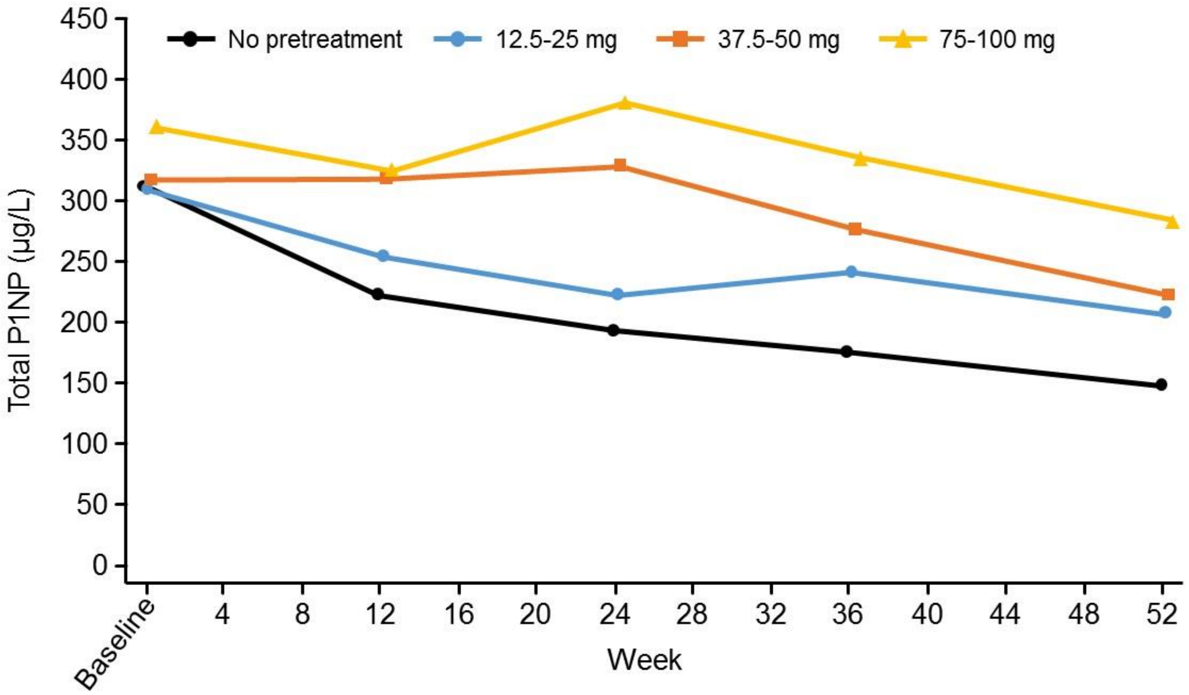

Data are shown as median.

Abbreviation: P1NP, procollagen type I intact N-terminal propeptide

Supplementary Figure S5: Changes in evocalcet dose stratified by pre-treatment cinacalcet dose

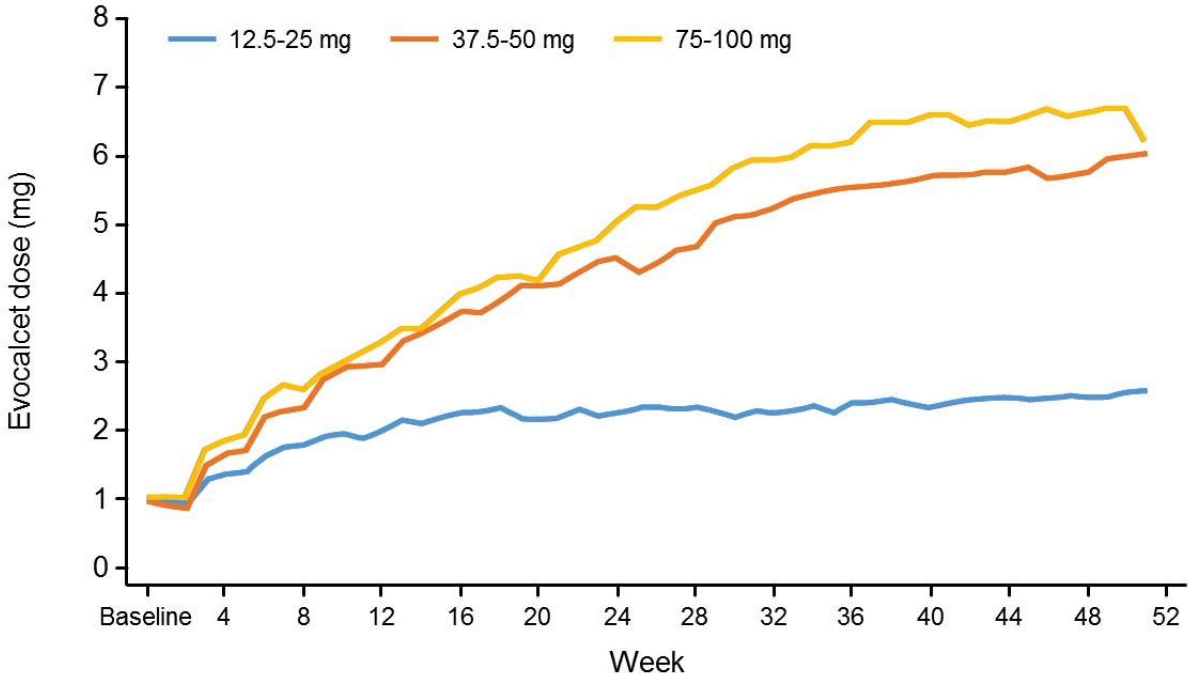

Supplementary Text S1: List of participating centers

Principal Investigator	Institution	Type of Review Board	Name of External Review Board
Yoshitaka Maeda	JA Toride Medical Center	External	Review Board of Human Rights and Ethics for Clinical Studies
Kazue Ueki	Sanshikai Toho Hospital	External	Review Board of Human Rights and Ethics for Clinical Studies
Takayuki Fujii	Seirei Sakura Citizen Hospital	External	Review Board of Human Rights and Ethics for Clinical Studies
Ryoichi Miyazaki	Fujita Memorial Hospital	External	Fukui General Hospital IRB
Hisanori Azekura	Sanaru Sun Clinic	External	Review Board of Human Rights and Ethics for Clinical Studies
Hirotake Kasuga	Kaikoukai Central Clinic	External	Nagoya Kyoritsu Hospital IRB
Yoshiyuki Tomiyoshi	Takagi Hospital	External	Fukuoka Sanno Hospital IRB
Takeaki Shinzato	Shinzato Clinic Urakami	External	Shin-Nihombashi Ishii Clinic IRB
Ryuji Iwashita	Ueyama Hospital	External	Koukeikai Sugiura Clinic IRB
Kenji Takada	Tsukuba Gakuen Hospital	External	Review Board of Human Rights and Ethics for Clinical Studies
Akio Suda	Suda Clinic	External	Review Board of Human Rights and Ethics for Clinical Studies
Takashi Nagaoka	Sagamihara Clinic	External	Shin-Nihombashi Ishii Clinic IRB
Mitsuru Yoshimoto	Ohno Memorial Hospital	Internal	
Masatomo Taniguchi	Fukuoka Renal Clinic	External	Tokyo Midtown Clinic IRB
Hiroshi Ogawa	Shinseikai Daiichi Hospital	External	Tokyo Midtown Clinic IRB

Supplementary Text S2: Study design and exclusion criteria

Study design

To maintain an iPTH concentration of 60–240 pg/mL, the following dose adjustment criteria determined whether evocalcet dosage was increased or decreased during the 52 weeks. The dose of evocalcet was increased to a maximum dose of 12 mg once daily by increments of 1 mg based on the following criteria: current dose was maintained for at least 3 weeks; iPTH concentration was >240 pg/mL; or if iPTH concentration was between 150–240 pg/mL, and the investigator determined that the dose could be increased; if the corrected serum calcium level was ≥8.4 mg/dL; and if the investigator deemed a dose escalation likely to be safe. The dose of evocalcet was generally reduced in 1-mg increments based on the following criteria: iPTH concentration was <60 pg/mL, or there was the presence of an adverse event that was determined to warrant an evocalcet dose reduction or suspension of treatment. Temporary suspension of treatment for up to four weeks was permitted, and if the patient was deemed unable to resume treatment, the subject's participation in the study was stopped. Treatment could be resumed at the same, or a lower, dose as that prior to withdrawal on the day of HD following the maximum interdialytic interval. Compliance with treatment was calculated as follows: adherence rate (%) = 100 × the number of days evocalcet was administered as prescribed / the total number of days of prescription.

Exclusion criteria (continued.)

Women who were pregnant, lactating, or of childbearing potential and prior participation in trial treatments including evocalcet were also other exclusion criteria. Candidates with a history of hypercalcemia, uncontrolled chronic medical conditions such as diabetes or hypertension, severe hepatic impairment (alanine transaminase or alanine transaminase ≥

100IU/L), severe drug allergy, a history of drug or alcohol abuse, a malignancy within 5 years of the first examination (excluding basal cell carcinoma or surgically resected cervical cancer), or a history of recent myocardial infarctions (New York Heart Association functional classification class III or higher) were excluded from the study. Patients deemed by the investigators to be unsuitable for the study for any other reason were also excluded.
